# Early-Life Risk Factors for Carotid Intima-Media Thickness and Carotid Stiffness in Adolescence

**DOI:** 10.1001/jamanetworkopen.2024.34699

**Published:** 2024-09-20

**Authors:** Isabelle A. van der Linden, Rozan Roodenburg, Sanne L. Nijhof, Cornelis K. van der Ent, Roderick P. Venekamp, Sabine E. I. van der Laan, Henk S. Schipper

**Affiliations:** 1 Department of Pediatric Pulmonology, Wilhelmina Children’s Hospital, University Medical Center Utrecht, Utrecht, the Netherlands; 2 Department of Pediatric Cardiology, Sophia Children’s Hospital, Erasmus Medical Center, Rotterdam, the Netherlands; 3 Department of Social Pediatrics, Wilhelmina Children’s Hospital, University Medical Center Utrecht, Utrecht, the Netherlands; 4 Julius Center for Health Sciences and Primary Care, Department of General Practice & Nursing Science, University Medical Center Utrecht, Utrecht University, Utrecht, the Netherlands

## Abstract

**Importance:**

Atherogenesis starts during childhood, making childhood and adolescence an important window of opportunity to prevent atherosclerotic cardiovascular disease later in life.

**Objective:**

To identify early-life risk factors for preclinical atherosclerosis in adolescence.

**Design, Setting, and Participants:**

This cohort study is part of the ongoing Wheezing Illness Study in Leidsche Rijn (WHISTLER) prospective birth cohort study, which includes 3005 healthy newborns born between December 2001 and December 2012 in the Leidsche Rijn area of Utrecht, the Netherlands. Eligible participants included those from the WHISTLER cohort who visited the clinic between March 2019 and October 2020 for adolescent follow-up. This study’s analyses were performed in January 2024.

**Exposures:**

Early-life growth was assessed at birth to 6 months, 5 years, and 12 to 16 years. Abdominal ultrasonography determined abdominal subcutaneous adipose tissue (SAT) and visceral adipose tissue (VAT) depth. Blood pressure (BP) percentiles and body mass index (BMI) *z* scores were used.

**Main Outcomes and Measures:**

Carotid ultrasonography was performed at age 12 to 16 years to assess carotid intima-media thickness (cIMT) and the distensibility coefficient (DC), established measures of preclinical atherosclerosis. Multivariable linear regression models were used to identify early-life risk factors for cIMT and DC in adolescence.

**Results:**

In total, 232 adolescents (median [IQR] age, 14.9 [13.7-15.8] years; 121 female [52.2%]) were included. More postnatal weight gain (*B* = 12.34; 95% CI, 2.39 to 22.39), higher systolic BP at 5 years (*B* = 0.52; 95% CI, 0.02 to 1.01), more VAT at 5 years (*B* = 3.48; 95% CI, 1.55 to 5.40), and a larger change in VAT between 5 and 12 to 16 years (*B* = 3.13; 95% CI, 1.87 to 4.39) were associated with a higher cIMT in adolescence. A higher BMI (*B* = −2.70, 95% CI,−4.59 to −0.80) and VAT at 5 years (*B* = −0.56; 95% CI, −0.87 to −0.25), as well as a larger change in BMI between 5 and 12 to 16 years (*B* = −3.63; 95% CI, −5.66 to −1.60) were associated with a higher carotid stiffness in adolescence. On the contrary, a larger change in SAT between 5 and 12 to 16 years (*B* = 0.37; 95% CI, 0.16 to 0.58) was associated with a higher carotid DC in adolescence.

**Conclusions and Relevance:**

In this cohort study of 232 participants, early-life growth parameters, and particularly abdominal VAT development, were associated with a higher cIMT and carotid stiffness in adolescence. These findings suggest that assessment of adipose tissue development during childhood can aid characterization of lifetime risk trajectories and tailoring of cardiovascular prevention and risk management strategies.

## Key Points

**Question:**

What are the early-life risk factors of preclinical atherosclerosis in adolescence?

**Findings:**

In this cohort study of 232 adolescents, more postnatal weight gain, higher systolic blood pressure, more abdominal visceral adipose tissue (VAT), and greater VAT increase in early life were associated with a greater carotid intima-media thickness in adolescence. A higher BMI, BMI increase, and VAT in early life were associated with enhanced carotid stiffness in adolescence.

**Meaning:**

These findings suggest that assessment of adipose tissue development during childhood can aid characterization of lifetime risk trajectories and tailoring of cardiovascular prevention and risk management strategies.

## Teaser

This cohort study analyzes the association of early-life characteristics with preclinical atherosclerosis, including carotid artery intima-media thickness and carotid stiffness, among adolescents in the Netherlands.

## Introduction

Atherosclerosis is a lipid-driven inflammatory disease. Major clinical manifestations include ischemic heart disease, ischemic stroke, and peripheral arterial disease, mainly affecting middle-aged and elderly people. Atherogenesis starts during childhood, making childhood and adolescence an important window of opportunity to prevent atherosclerotic cardiovascular disease (CVD) later in life.^1^

Atherogenesis in the young is accelerated by modifiable risk factors such as obesity, hypertension, hyperlipidemia, and hyperglycemia.^2^ The prevalence of childhood overweight and obesity have surged over the last few decades.^3^ Multiple birth cohort studies have established a negative association of body mass development in children with obesity and overweight with CVD development later in life.^4,5,6^ Already in childhood, negative associations of adiposity with the vascular system have been reported.^7,8,9^ In adults, a more atherogenic role for visceral adipose tissue (VAT) compared with subcutaneous adipose tissue (SAT) is established, which can partially be explained by the higher release of inflammatory adipokines and lipids from VAT into the circulation.^10^ The question remains if and to what extent VAT and SAT already play an atherogenic role in childhood.

Carotid artery intima-media thickness (cIMT) is a marker of preclinical atherosclerosis and can independently project cardiovascular events in adults.^11^ cIMT is a commonly used noninvasive measurement in pediatrics because exposure to cardiovascular risk factors during childhood is associated with increased cIMT in adolescence and cardiovascular events in adults.^12,13,14,15,16,17^ Another preclinical marker for atherosclerosis is increased carotid arterial stiffness, which is associated with future CVD and all-cause mortality in adults.^18^ The distensibility coefficient (DC) is one of the stiffness parameters for which pediatric reference values are available.^19^ Stiffness of the arterial wall may occur in early atherogenesis before structural wall changes become detectable, which makes DC an interesting marker for preclinical atherosclerosis in children and adolescents.

The main aim of this study is to identify early-life risk factors for preclinical atherosclerosis in adolescence as assessed by cIMT and the carotid DC in a longitudinal birth cohort. More specifically, this study focuses on early-life VAT development as a potentially relevant yet unexplored risk factor for early atherogenesis.

## Methods

### Study Design and Population

This cohort study was approved by the medical ethics review committee of the University Medical Center Utrecht and followed the Strengthening the Reporting of Observational Studies in Epidemiology (STROBE) reporting guideline. We obtained data from the Wheezing Illness Study in Leidsche Rijn (WHISTLER), a large prospective population-based birth cohort study on risk factors for wheezing illnesses. Participants and their parents or legal guardians provided active written informed consent. Study design and rationale were described in detail elsewhere.^20^ Briefly, healthy infants born in Leidsche Rijn, a new residential area near the city of Utrecht in the Netherlands, were enrolled at the age of 2 or 3 weeks. Exclusion criteria were gestational age less than 36 weeks, major congenital abnormalities, and neonatal respiratory disease. In 2007, the study was extended for cardiovascular research questions. The WHISTLER study recruited 3005 infants between 2001 and 2012, of whom 2784 completed the baseline examination.^21^ For the WHISTLER follow-up wave at 5 years of age, a core cohort of 1000 children was reassessed.^22^ In 2019, the WHISTLER adolescents (age 12-16 years) follow-up wave was initiated as the basis for the current study. Participants were contacted multiple times by telephone as well as by email to attain greater follow-up numbers. However, follow-up of this wave was paused multiple times due to the COVID-19 pandemic, interrupting progression of the follow-up wave. In October 2020, we ultimately stopped the WHISTLER adolescents follow-up wave. Additionally, an estimated 23% of participants were unreachable due to changes in contact information, relocation, or declined participation based on analysis of a subset of participants. In the current study, we focused on 3 developmental phases: birth to 6 months, early childhood (5 years), and adolescence (12-16 years).

### Measures

#### Growth Parameters

Growth parameters were measured at birth, 6 months, 5 years, and 12 to 16 years, as specified earlier.^20^ For postnatal weight gain, *z* scores for weight at birth (corrected for gestational age and sex) and weight at 6 months (corrected for age and sex) were calculated based on Dutch growth reference charts.^23^ Postnatal weight gain was assessed by the difference in weight *z* score between birth and 6 months. Body mass index (BMI; calculated as weight in kilograms divided by height in meters squared) at 5 years, BMI at 12 to 16 years, and thereby change in BMI, were also corrected for sex and age using the same Dutch growth reference chart tool.^23^ Change in BMI is the difference in BMI *z* score between 5 years and 12 to 16 years.^23^

#### Abdominal Adipose Tissue Measurements

Ultrasonography has proven to be a reliable tool to assess abdominal VAT and SAT.^24^ VAT and SAT depth were measured in millimeters as previously described for the WHISTLER population at the age of 5 years.^9^ In adolescence, abdominal fat was measured using the MyLabOne Ultrasound 8100 with a SC-3421 convex transducer (Esaote). For VAT depth, the mean distance from the 3 different angles was taken for further analysis. For SAT depth, the mean of 3 measurements was used for analysis. In addition, the differences in abdominal fat measurements between 5 years and 12 to 16 years were used for analyses (change in SAT and change in VAT).

#### Blood Pressure

Blood pressure (BP) was measured on the right arm using an automatic oscillometric device. At least 2 measurements were taken, and if a difference of 10 or more mmHg was found, a third measurement was performed. A BP percentile was calculated from the lowest BP value, based on sex, height, and age using the 2017 American Academy of Pediatrics Guidelines for Screening and Management of High Blood Pressure.^25^ This BP percentile was used in the regression analyses.

#### Questionnaire

An online questionnaire among adolescents inquired about smoking status. A separate questionnaire among one of the parents specifically asked whether the parents or grandparents of the participant experienced CVD before the age of 60 years.

#### Outcome Measures

Vascular properties of the right common carotid artery were measured in adolescence (12-16 years), using the ART.LAB program (Esaote).^26^ The ART.LAB program was coupled with an Esaote MylabOne sonographer equipped with a SL3323 linear probe (4.0-13.0 MHz). Intima-media thickness was measured on 128 radiofrequency lines (brightness-mode; up to a 4-cm zone) and carotid distension and diameter on 14 radiofrequency lines (frequency bandwidth–mode) in real time. Measurements were performed with research participants in a supine position with their heads turned 45° to the contralateral side of the artery after at least 10 minutes of resting. The measurements were conducted according to the Mannheim Intima-Media Thickness Consensus.^27^ This included measuring longitudinal and perpendicular to the ultrasonography beam in lateral view at least 5 mm proximal to the bifurcation and in an area with clearly defined lumen-intima. One brightness-mode file and one frequency bandwidth–mode file were acquired per participant (6 beats collected per file), with the carotid diameter calculated as the mean of B-mode and FB-mode diameters. cIMT and the DC were then automatically calculated by the program. cIMT and carotid distension were measured 3 times, after which a mean was calculated. The DC reflects the absolute change in vessel diameter during systole for a given pressure change.^28^ The formula follows: DC = (∆A/A)/(∆P), where A is the diastolic lumen area, ∆A is the change in cross-sectional area from diastole to systole, and ∆P is the associated change in local blood pressure.

### Statistical Analysis

#### Descriptive Statistics

Participant characteristics were described based on original, nonimputed data. Numeric variables are expressed as mean and SD when normally distributed or median and IQR if not normally distributed. Categorical variables are described as frequencies and percentages. Selection bias was analyzed using independent *t* tests comparing the characteristics at birth of WHISTLER participants that did participate in the current study with the WHISTLER participants that did not (eTable 1 in Supplement 1).

#### Missing Data

The percentage of missing values across the 14 factors varied between 0% and 47% (eFigure 1 in Supplement 1), with 0% and 6% missing data for the outcome variables cIMT and carotid DC, respectively. In total, 135 case records (58%) were incomplete. Multiple imputation was used to create and analyze 60 multiply imputed datasets with 50 iterations to improve accuracy and statistical power.^29^ Incomplete variables were imputed under fully conditional specification, using the Markov Chain Monte Carlo method. Predictive mean matching was used because some of the variables were not normally distributed.^30^ Next, the 60 imputed datasets were pooled into one final dataset based on mean aggregation.

#### Multivariable Linear Regression Analyses

After multiple imputation, multivariable linear regression models with stepwise backward removal were created to identify possible early-life risk factors for cIMT and carotid distensibility in adolescence out of the following candidate variables: birth weight, postnatal weight gain, sex, age, family history of CVD, systolic and diastolic BP, BMI, VAT, SAT, change in BMI, change in VAT, and change in SAT. We chose these variables based on clinical knowledge and current literature. At each step, variables were added based on *P* values, and a *P* value threshold of .10 was used to set a limit on the total number of variables included in the final model. In the end, the model with the highest adjusted *R^2^* was selected as the final model. We checked if multicollinearity existed by calculating the variance inflation factor. The threshold for statistical significance was a 2-tailed *P* < .05. All analyses were performed using SPSS Statistics version 26.0 (IBM). Analyses for the current study were performed in January 2024.

## Results

### Participant Characteristics

In total, 232 adolescents (median [IQR] age, 14.9 [13.7-15.8] years; 121 female [52.2%]) were included. They were born with a median (IQR) gestational age of 40.0 (39.0-41.0) weeks, a mean (SD) birth weight of 3530 (506) g, and showed a mean (SD) postnatal weight gain *z* score of 0.2 (0.9) between birth and 6 months. Early-life characteristics included a mean (SD) BMI *z* score of −0.2 (1.1), a mean (SD) systolic BP percentile of 72.2 (20.0), and a mean (SD) diastolic BP percentile of 38.0 (22.8). In adolescence, we found a mean (SD) BMI *z* score of 0.2 (1.1), a mean (SD) systolic BP percentile of 45.5 (24.2), and a mean (SD) diastolic BP percentile of 18.7 (15.9). These results are similar to other studies in healthy peers.^25,31^ Mean (SD) early-life abdominal VAT was 35.6 (6.3) mm, and median (IQR) SAT depth was 5.9 (4.7-4.6) mm. Median (IQR) adolescent abdominal VAT was 43.4 (37.7-51.5) mm and median (IQR) SAT depth was 11.9 (8.6-18.6) mm. Based on international age- and sex-specific BMI cutoff values for obesity, none of the participants had obesity in early life and 5 participants had obesity in adolescence.^32^ Furthermore, 16 participants had an SBP in the 95th percentile or greater in early life and 5 participants had an SBP in the 95th percentile or greater in adolescence.^25^ Median (IQR) adolescent cIMT was 432 (392-491) μm and median (IQR) carotid DC was 40 × 10^−3^ kPa (38 × 10^−3^ kPa to 50 × 10^−3^ kPa). Participant characteristics are further described in Table 1 and stratified by sex in eTable 2 in Supplement 1. Because only 2 adolescents reported smoking, this variable was excluded from further analysis. The risk of multicollinearity was considered low, with variance inflation factor values ranging from 1.13 to 2.16. Participants included in the WHISTLER adolescents follow-up wave showed similar characteristics at birth as those not participating in the adolescents follow-up wave in terms of sex, gestational age, birth weight, parental BMI, maternal smoking during pregnancy, and socioeconomic status (eTable 1 in Supplement 1).

**Table 1.** Participant Characteristics

Characteristic	Birth (0-6 mo)		Early life (5 y)		Adolescence (12-16 y)	
	Outcome	Participants, No.^a^	Outcome	Participants, No.^b^	Outcome	Participants, No.^c^
Gestational age, median (IQR), wk	40.0 (39.0-41.0)	225	NA	NA	NA	NA
Birth weight, mean (SD), g	3530 (506)	225	NA	NA	NA	NA
Postnatal weight gain, mean (SD), *z* score^d^	0.2 (0.9)	187	NA	NA	NA	NA
Sex, No. (%)						
Female	NA	NA	123 (53.0)	232	121 (52.2)	232
Male	NA	NA	109 (47.0)	232	111 (47.8)	232
Age, median (IQR), y	NA	NA	5.3 (5.2-5.5)	199	14.9 (13.7-15.8)	232
BMI, mean (SD)^e^	NA	NA	15.1 (1.2)	199	19.8 (3.1)	232
BMI, mean (SD), *z *score	NA	NA	−0.2 (1.1)	199	0.2 (1.1)	232
Smoking, No. (%)	NA	NA	NA	NA	2 (0.9)	223
Family history of cardiovascular disease, No. (%)	NA	NA	NA	NA	76 (33.5)	227
Systolic BP, median (IQR), mm Hg	NA	NA	102 (97-106)	197	109 (103-114)	231
Systolic BP, mean (SD), percentile^f^	NA	NA	72.2 (20.0)	197	45.5 (24.2)	231
Diastolic BP, mean (SD), mm Hg	NA	NA	53.8 (7.0)	197	56.7 (6.4)	231
Diastolic BP, mean (SD), percentile^f^	NA	NA	38.0 (22.8)	197	18.7 (15.9)	231
SAT, median (IQR), mm	NA	NA	5.9 (4.7-7.6)	188	11.9 (8.6-18.6)	185
∆SAT, median (IQR), mm^g^	NA	NA	NA	NA	5.3 (3.3-11.0)	153
VAT, median (IQR), mm	NA	NA	35.6 (6.3)	156	43.4 (37.7-51.5)	180
∆VAT, mean (SD), mm^g^	NA	NA	NA	NA	9.6 (10.7)	124
Carotid intima-media thickness, median (IQR), μm	NA	NA	NA	NA	432 (392-491)	232
Distensibility coefficient, median (IQR), 10^−3^ kPa	NA	NA	NA	NA	40 (38-50)	218

Abbreviations: BMI, body mass index; BP, blood pressure; NA, not applicable; SAT, abdominal subcutaneous adipose tissue depth; VAT, abdominal visceral adipose tissue depth.

^a^ Denominator of birth characteristics.

^b^ Denominator of early-life characteristics.

^c^ Denominator of adolescent characteristics.

^d^ Postnatal weight gain measured as the difference in weight *z* score from birth (corrected for sex and gestational age) to 6 months of age (corrected for sex and age).

^e^ BMI was calculated as weight in kilograms divided by height in meters squared.

^f^ BP percentiles were calculated based on sex, height, and age.

^g^ ∆ is the difference between the adolescence (12-16 years) and early-life (5 years) value.

### Risk Factors for cIMT in Adolescence

Higher postnatal weight gain (*B* = 12.34; 95% CI, 2.39-22.39; *P* = .02), higher systolic BP (*B* = 0.52; 95% CI, 0.02-1.01; *P* = .04), higher VAT (*B* = 3.48; 95% CI, 1.55-5.40; *P* < .001), and a larger change in VAT between early life and adolescence (*B* = 3.13; 95% CI, 1.87-4.39; *P* < .001) were associated with a higher cIMT in adolescence (Table 2 and eFigure 2 in Supplement 1). For each 1 SD increase in postnatal weight gain, systolic BP, VAT, and change in VAT, adolescent cIMT increased by 11.7 μm, 9.5 μm, 18.3 μm, and 25.6 μm, respectively.

**Table 2.** Multivariable Linear Regression for the Dependent Variable Carotid Intima-Media Thickness in Adolescence^a^

Risk factor	*B* (95% CI)	*P* value
Postnatal weight gain^b^	12.34 (2.39 to 22.39)	.02
Early-life systolic blood pressure^c^	0.52 (0.02 to 1.01)	.04
Early-life VAT	3.48 (1.55 to 5.40)	<.001
∆VAT^d^	3.13 (1.87 to 4.39)	<.001
Sex	11.36 (−7.58 to 30.31)	.24
Family history of cardiovascular disease	−13.08 (−32.73 to 6.57)	.19

Abbreviation: VAT, abdominal visceral adipose tissue depth.

^a^ Model includes carotid intima-media thickness in adolescence (adjusted *R*^2^ = 0.11; *F* = 5.68; *P* < .001). Excluded risk factors after stepwise elimination included change in BMI, adolescent age, early-life age, change in abdominal subcutaneous adipose tissue depth, early-life diastolic blood pressure, early-life abdominal subcutaneous adipose tissue depth, early-life body mass index and birth weight. The analysis was conducted with imputed data.

^b^ Postnatal weight gain was measured as the difference in weight z score from birth to 6 months of age (corrected for age and sex).

^c^ Blood pressure percentiles were calculated based on sex, height, and age.

^d^ ∆ is the difference between the adolescence (12-16 years) and early-life (5 years) value.

### Risk Factors for Carotid Distensibility in Adolescence

Higher early-life BMI (*B* = −2.70; 95% CI, −4.59 to −0.80; *P* = .006), higher VAT (*B* = −0.56; 95% CI, −0.87 to −0.25; *P* < .001), lower change in SAT (*B* = 0.37; 95% CI, 0.16 to 0.58; *P* = .001), and higher change in BMI from early life to adolescence (*B* = −3.63; 95% CI, −5.66 to −1.60; *P* = .001) were associated with a lower adolescent DC, which indicates a higher vascular stiffness (Table 3 and eFigure 2 in Supplement 1). For each 1 SD increase in early-life BMI, early-life VAT, and change in BMI between early life and adolescence, adolescent carotid DC decreased by 2.7 × 10^−3^ kPa, 3.0 × 10^−3^ kPa and 3.3 × 10^−3^ kPa, respectively. On the contrary, each 1 SD increase in change in SAT was associated with an increase in adolescent carotid DC of 3.1 × 10^−3^ kPa.

**Table 3.** Multivariable Linear Regression for the Dependent Variable Carotid Distensibility in Adolescence^a^

Risk factor	*B* (95% CI)	*P* value
Postnatal weight gain^b^	1.30 (−0.25 to 2.85)	.10
Early-life BMI	−2.70 (−4.59 to −0.80)	.006
Early-life VAT	−0.56 (−0.87 to −0.25)	<.001
∆VAT^c^	−0.18 (−0.38 to 0.02)	.07
Early-life SAT	−0.60 (−1.29 to 0.08)	.08
∆SAT^c^	0.37 (0.16 to 0.58)	.001
∆BMI^c^	−3.63 (−5.66 to −1.60)	.001
Sex	1.78 (−1.21 to 4.78)	.24
Family history of cardiovascular disease	−2.07 (−5.17 to 1.04)	.19

Abbreviations: BMI, body mass index; SAT, abdominal subcutaneous adipose tissue depth; VAT, abdominal visceral adipose tissue depth.

^a^ Model includes carotid distensibility coefficient in adolescence (adjusted *R*^2^ = 0.13; *F* = 4.92; *P* < .001). Excluded factors after stepwise elimination included early-life diastolic blood pressure, birth weight, early-life systolic blood pressure, early-life age, and adolescent age. The analysis was conducted with imputed data.

^b^ Postnatal weight gain was measured as the difference in weight *z* score from birth to 6 months of age (corrected for age and sex).

^c^ ∆ is the difference between the adolescence (12-16 years) and early-life (5 years) value.

## Discussion

The aim of this cohort study was to establish early-life risk factors for preclinical atherosclerosis in adolescence, as assessed by cIMT and carotid distensibility. The present study showed that more postnatal weight gain, a higher systolic BP, more abdominal VAT, and a higher VAT increase in early life were associated with a thicker cIMT in adolescence (eFigure 2 in Supplement 1). A higher BMI, BMI increase, and VAT in early life were associated with a stiffer carotid in adolescence (eFigure 2 in Supplement 1). These findings suggest that exposure to these early-life factors induces lasting atherogenic changes in the arterial system. Moreover, our findings underscore the atherogenic role of abdominal VAT from early life onwards. The main risk factors will be discussed in depth per section, starting with early-life VAT as a mutual risk factor for cIMT and DC, whereafter risk factors for cIMT and DC will be reviewed in order of effect size (eFigure 2 in Supplement 1).

Early-life abdominal VAT was identified as a key adverse factor associated with cIMT and carotid stiffness in adolescence. So far, pediatric studies show varying results regarding the association of abdominal VAT with cardiometabolic risk in childhood.^33,34,35,36,37,38,39,40,41,42,43,44,45,46^ Most cross-sectional studies report an adverse association of VAT with cardiometabolic risk,^34,36,39,40,41,42,43,44,45^ but others do not demonstrate any association.^38,46^ These inconsistent findings may be due to differences in sample size, outcome measures, age group, and inclusion of participants with obesity. Previous studies used different VAT measuring techniques, such as dual-energy x-ray absorptiometry,^33,39,45^ ultrasonography,^44^ bioelectrical impedance analysis,^47,48,49^ computed tomography,^37,39,41,43^ magnetic resonance imaging,^38,42,46,50,51^ or proxies for VAT.^36,47,48,49,52^ More homogeneous results emerge when early-life abdominal obesity is associated with preclinical atherosclerosis measures. A large cohort of 4709 healthy adolescents and young adults^47^ found that various measures of abdominal obesity were associated with a higher cIMT and carotid stiffness, longitudinally as well as cross-sectionally. The added value of the current study is identification of abdominal VAT as a risk factor, rather than abdominal obesity in general. The WHISTLER study previously demonstrated that increased abdominal VAT was cross-sectionally associated with increased cIMT and lower carotid wall distensibility at 5 years of age.^9^ Pediatric studies using proxies for VAT, such as waist circumference, report associations with an increased cIMT^9,47,48,49,52^ and decreased carotid distensibility.^9,47^ Considering these findings, abdominal VAT measurements may provide important clues for the identification of children with an adverse cardiovascular risk profile with implications for pediatric screening strategies.

Another factor associated with adolescent cIMT was postnatal weight gain, as previously reported in younger children within the WHISTLER population.^21^ However, no association of postnatal weight gain with adolescent carotid distensibility was seen. Most studies investigating the association of early-life growth with cardiovascular outcomes have been performed in premature or small-for-gestational age participants, leaving the impact within a healthy population understudied.^53,54^ Our findings are in line with reports from childhood and adult studies that show an adverse association of postnatal catch-up growth with cardiovascular risk factors.^21,55,56,57^ Excessive early weight gain may increase the risk of developing cardiovascular risk factors,^21^ just as it does after fetal growth restriction.^58^ However, the underlying mechanisms by which excessive early-life weight gain leads to the development of cardiovascular risk factors are still largely unknown, which cautions extrapolating these findings to postnatal growth interventions.

We reported BP values within the normal range and found that systolic BP was positively associated with adolescent cIMT.^25^ This finding is in line with an earlier study^59^ reporting that prehypertension is associated with a higher cIMT in a population aged 10 to 23 years, which suggests that BP influences vascular remodeling in the pediatric population at a nonhypertensive level and might be explained by adaptive changes of the media layer, rather than atherosclerotic changes in the intima layer.^60^ Notably, BP was not associated with carotid DC because carotid DC reflects the absolute change in carotid diameter during systole for a given BP change.^28^ In other words, carotid DC already accounts for BP changes.

Key factors associated with adolescent carotid DC included a higher early-life BMI, change in BMI, early-life VAT, and change in SAT. The association with BMI is in line with earlier cross-sectional studies in adolescents,^61,62^ and may be explained by carotid hemodynamics. Carotid distension is partly determined by cardiac output, which is increased in adolescents with a higher BMI.^63^ This hemodynamic effect may account for the identification of early-life BMI and change in BMI as factors associated with carotid DC, rather than cIMT. Moreover, fundamental studies have shown that a higher BMI can lead to increased arterial stiffness via several mechanisms, including collagen accumulation in the arterial wall and stiffness of endothelial and vascular smooth muscle cells.^64,65^ In contrast with a higher BMI and early-life VAT, higher change in SAT was associated with reduced carotid stiffness in adolescence. A protective effect of SAT for cardiovascular health has been suggested in previous studies^51,66,67,68^ and is supported by more fundamental research showing an increased production of vasoprotective and anti-inflammatory adipokines by SAT, such as adiponectin.^69^ In adult studies, it is well documented that VAT confers a higher cardiometabolic risk compared with SAT, but this is not yet clear in children.^70,71^ Considering the rapid change in body composition and rising prevalence of obesity in childhood, elucidating the dynamics of adipose tissue development, and its association with CVD outcomes is an important topic for future research.

Finally, we endeavored to reflect on our findings from a physiological perspective of vascular aging. Normal aging is associated with thickening of the arterial walls and an increase in arterial stiffness. Generally, our study found cIMT and DC values within healthy reference ranges for age.^19,47,72^ According to these reference data, cIMT increases by approximately 3 μm and DC decreases by approximately 2 × 10^−3^ kPa per year in children aged 5 to 16 years. Extrapolating this to the current study, our data suggests that a 1 SD increase in early-life VAT reflects approximately 6 years of vascular aging by cIMT, and a 1 SD increase in change in VAT reflects approximately 8.5 years of vascular aging by cIMT. Likewise, a 1 SD increase in early-life BMI, VAT, or change in BMI reflects approximately 1.5 years of vascular aging measured by DC. This accelerated progression of vascular aging is likely to be of clinical relevance, and longitudinal studies with a larger follow-up time are needed to establish relevance at a later age.

### Strengths and Limitations

The main strength of this study lies in its prospective longitudinal design with assessment of relevant early-life parameters at different ages. To our knowledge, this is the first study that evaluates the relevance of abdominal VAT and SAT in childhood for preclinical atherosclerosis in adolescence. Consistent measurements of abdominal fat were performed in childhood and adolescence using similar protocols, and trained staff collecting the data. Furthermore, training in carotid ultrasonography was carried out every 6 months. These efforts and the use of standard operating procedures for all physical measurements were aimed at minimizing interobserver variation.

This study also has limitations. By applying the family history of CVD questionnaire, recall bias might exist. The studied population was recruited from a new and upcoming district in the Netherlands (Leidsche Rijn, Utrecht), with a predominantly rich, White, and highly educated population. Consequently, results of this study may not be generalizable to populations with lower income, education, or different ethnicity. Because the incidence of obesity and hypertension was low in this healthy birth cohort, we do not expect major effects on the outcome measures in this cohort. Another consideration is the study’s missing data, which we tried to minimize using aggregated multiple imputation methods. However, potential loss of information on the variation due to missing data remains. Because a stepwise backward removal method was used, it is important to mention the possible overestimation of *P* values in the final model and reduced performance when projecting cIMT and carotid stiffness in a new sample. Additionally, other factors such as diet and physical activity might be influential factors for cardiovascular outcome measures such as cIMT and the DC. Earlier studies already showed that physical activity and dietary intake are associated with cIMT at 17 years.^60^ This data was unfortunately not available in this cohort but is relevant to take into account in future research.

Given the diversity of risk factors for cIMT and carotid distensibility, it appears that cardiovascular risk development is multifactorial and that one type of atherosclerotic measure does not fully capture the atherosclerotic risk in this population. Several experts in the field have in fact advocated a multimodal and multisite approach to capture the complexity of atherosclerosis as a systemic disease.^73,74^ Because atherogenesis starts in the iliac arteries and abdominal aorta during childhood, and is later seen in higher regions of the arterial tree, preclinical measures of atherosclerosis in the aorta could advance the assessment of preclinical atherosclerosis in pediatric studies as an addition to cIMT and DC measurements.^75,76^

## Conclusions

The present cohort study showed that postnatal weight gain, systolic BP, abdominal VAT, and abdominal VAT increase in early life were associated with a thicker cIMT in adolescence. Furthermore, BMI, BMI increase, and abdominal VAT in early life were associated with a stiffer carotid in adolescence, while an increase in abdominal SAT was associated with reduced carotid stiffness in adolescence. These findings underscore the potential atherogenic role of visceral adipose tissue from early life onwards. Assessment of adipose tissue development during childhood can aid characterization of lifetime risk trajectories and tailoring of cardiovascular prevention and risk management strategies.

## Notes

Supplement 1. **eTable 1.** Comparison of Participant Characteristics Between Participants Who Attended This Adolescent Follow-Up and Who Did Not Attend This Follow-Up **eFigure 1.** Missing Data **eTable 2.** Participant Characteristics Stratified by Sex **eFigure 2.** Early Life Risk Factors for Carotid Intima-Media Thickness and Carotid Distensibility in Adolescence

Supplement 2.
Data Sharing Statement
