# Supplementary material for: Early-Life Risk Factors for Carotid Intima-Media Thickness and Carotid Stiffness in Adolescence
Source: JAMA Netw Open. 2024 Sep 20;7(9):e2434699. doi: 10.1001/jamanetworkopen.2024.34699 (PMC11415786; doi:10.1001/jamanetworkopen.2024.34699)
Supplement: Supplement 1. — eTable 1. Comparison of Participant Characteristics Between Participants Who Attended This Adolescent Follow-Up and Who Did Not Attend This Follow-Up eFigure 1. Missing Data eTable 2. Participant Characteristics Stratified by Sex eFigure 2. Early Life Risk Factors for Carotid Intima-Media Thickness and Carotid Distensibility in Adolescence [file jamanetwopen-e2434699-s001.pdf]

Supplemental Online Content

van der Linden IA, Roodenburg R, Nijhof SL, et al. Early-life risk factors for carotid intima-media thickness and carotid stiffness in adolescence. *JAMA Netw. Open*. 2024;7(9):e2434699. doi:10.1001/jamanetworkopen.2024.34699

eTable 1. Comparison of Participant Characteristics Between Participants Who Attended This Adolescent Follow-Up and Who Did Not Attend This Follow-Up
eFigure 1. Missing Data
eTable 2. Participant Characteristics Stratified by Sex
eFigure 2. Early-Life Risk Factors for Carotid Intima-Media Thickness and Carotid Distensibility in Adolescence

This supplemental material has been provided by the authors to give readers additional information about their work.

© 2024 van der Linden IA et al. *JAMA Network Open.*

eTable 1. Comparison of participant characteristics between participants who attended this adolescent follow-up and who did not attend this follow-up

Characteristic No. (%) or mean (SD)	No adolescent follow-up (N=2773)	N[a]	Adolescent follow-up (N=232)	N[b]	P	95% CI
Sex, female	1284 (50%)	2556	122 (54%)	228	0.34	-0.10 – 0.04
Birth weight, g	3542 (503)	2488	3530 (506)	225	0.74	-56.86 – 80.41
GA, wks	39.4 (1.4)	2491	39.6 (1.4)	225	0.20	-0.30 – 0.06
Maternal BMI	24.7 (4.0)	2109	24.7 (4.0)	205	0.99	-0.57 – 0.58
Paternal BMI	25.1 (3.2)	2002	24.9 (3.1)	194	0.24	-0.19 – 0.74
Maternal smoking in pregnancy	157 (6.3%)	2482	15 (6.7%)	225	0.84	-0.04 – 0.03
Maternal ever smoked	712 (34.5%)	2064	72 (35.6%)	202	0.74	-0.06 – 0.08
Paternal ever smoked	758 (40.5%)	1870	85 (45.7%)	186	0.17	-0.02 – 0.13
Maternal SES, high educated	1438 (69.7%)	2063	134 (65.7%)	204	0.24	-0.03 – 0.11
Paternal SES, high educated	1253 (64.0%)	1957	113 (59.8%)	189	0.25	-0.03 – 0.11
Maternal ethnicity, Caucasian	1695 (81.6%)	2077	177 (87.6%)	202	0.03*	-0.12 - -0.01

GA: Gestational age, BMI: Body Mass Index in kg/m^2, SES: Socioeconomic status. [a]Denominator of WHISTLER participants who did not attend adolescent follow-up. [b]Denominator of WHISTLER participants who did attend adolescent follow-up.

© 2024 van der Linden IA et al. *JAMA Network Open.*

eFigure 1. Missing Data

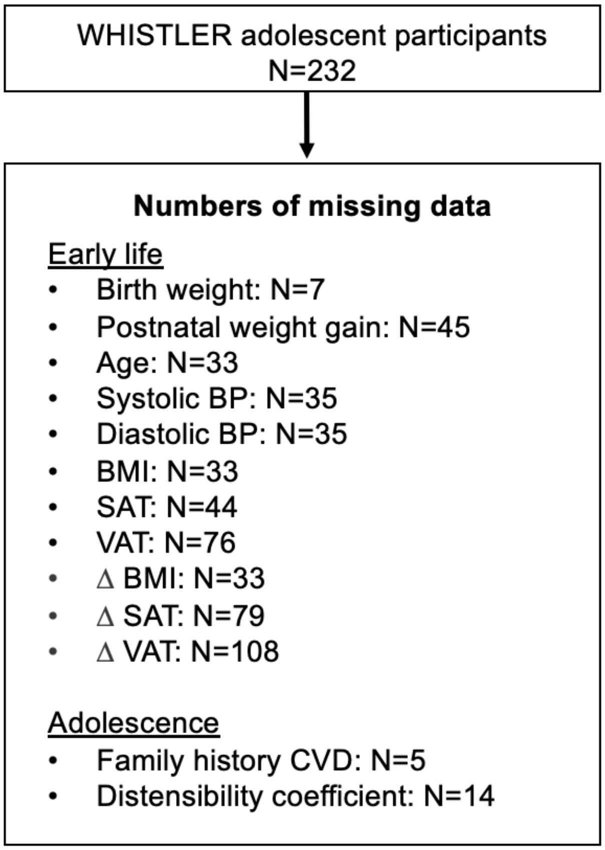

Postnatal weight gain: measured as the difference in weight Z-score from birth (corrected for sex and gestational age) to 6 months of age (corrected for age and sex). cIMT: carotid intima-media thickness, BMI: Body Mass Index, BP: blood pressure, SAT: abdominal subcutaneous adipose tissue depth, VAT: abdominal visceral adipose tissue depth, CVD: cardiovascular disease. "∆" is the difference between adolescence (12-16 years) and early life (5 years) value.

© 2024 van der Linden IA et al. *JAMA Network Open.*

eTable 2. Participant characteristics stratified by sex (*part 1*)

Characteristic	Birth (0-6 months)				N[a]
	Male	N[a]	Female	N[a]	Total
Gestational age, wks	39.0 (39.0–40.8)	104	40.0 (39.0–41.0)	121	225
Birth weight, g	3570 (530)	104	3495 (484)	121	225
Postnatal weight gain, Z-score[d]	0.2 (1.1)	82	0.0 (0.9)	105	187
Age, y	NA				
BMI, kg/m^2	NA				
BMI, Z-score	NA				
Smoking, No. (%)	NA				
Family history of CVD, No. (%)	NA				
Systolic BP, mmHg	NA				
Percentile[e]	NA				
Diastolic BP, mmHg	NA				
Percentile[e]	NA				
SAT, mm	NA				
∆SAT, mm	NA				
VAT, mm	NA				
∆VAT, mm	NA				
cIMT, µm	NA				
Distensibility coefficient, 10^{-3}*/ kPa*	NA				

This is original non-imputed data. Shown are number (N) (%), mean (SD) for variables with normal distributions or median (interquartile range) for variables with skewed distributions. BP: blood pressure, BMI: Body Mass Index, SAT: abdominal subcutaneous adipose tissue depth, VAT: abdominal visceral adipose tissue depth, CVD: cardiovascular disease, cIMT: carotid intima-media thickness, "∆" is the difference between adolescence (12-16 years) and early life (5 years) value. [a]Denominator of birth characteristics. [b]Denominator of early life characteristics. [c]Denominator of adolescent characteristics. [d]Postnatal weight gain measured as the difference in weight Z-score from birth (corrected for sex and gestational age) to 6 months of age (corrected for sex and age). [e]BP percentiles were calculated based on sex, height, and age.

© 2024 van der Linden IA et al. *JAMA Network Open.*

eTable 2. Participant characteristics stratified by sex (continued - *part 2*)

Characteristic	Early life (5 years)				N[b]
	Male	N[b]	Female	N[b]	Total
Gestational age, wks	NA				
Birth weight, g	NA				
Postnatal weight gain, Z-score[d]	NA				
Age, y	5.3 (5.2–5.5)	91	5.3 (5.2–5.5)	108	199
BMI, kg/m^2	15.2 (1.2)	91	15.0 (1.2)	108	199
BMI, Z-score	-0.2 (1.1)	91	-0.2 (1.0)	108	199
Smoking, No. (%)	NA				
Family history of CVD, No. (%)	NA				
Systolic BP, mmHg	104 (99–110)	89	104 (99–108)	108	197
Percentile[e]	70.3 (22.2)	89	73.8 (17.9)	108	197
Diastolic BP, mmHg	54.6 (6.6)	89	53.0 (7.4)	108	197
Percentile[e]	40.4 (23.3)	89	35.9 (22.2)	108	197
SAT, mm	5.4 (4.2–6.8)	85	6.5 (5.3–8.3)	103	188
ΔSAT, mm	NA				
VAT, mm	36.2 (5.6)	76	35.0 (6.8)	80	156
ΔVAT, mm	NA				
cIMT, µm	NA				
Distensibility coefficient, 10^{-3}/ kPa	NA				

Characteristic	Adolescence (12-16 years)				N[c]
	Male	N[c]	Female	N[c]	Total
Gestational age, wks	NA				
Birth weight, g	NA				
Postnatal weight gain, Z-score[d]	NA				
Age, y	14.7 (13.4–15.7)	109	14.9 (13.9–15.9)	123	232
BMI, kg/m^2	19.4 (3.3)	109	20.2 (2.9)	123	232
BMI, Z-score	0.1 (1.3)	109	0.3 (1.0)	123	232
Smoking, No. (%)	2 (1.8)	102	0 (0.0)	121	223
Family history of CVD, No. (%)	32 (29.4)	104	44 (35.8)	123	227
Systolic BP, mmHg	112.8 (106–119.9)	108	111 (105–116)	123	231
Percentile[e]	44.5 (25.7)	108	46.4 (22.9)	123	231
Diastolic BP, mmHg	56.7 (6.7)	108	56.7 (6.2)	123	231
Percentile[e]	20.6 (16.3)	108	17.0 (15.4)	123	231
SAT, mm	9.3 (7.2– 15.3)	80	13.7 (10.1–20.0)	105	185
ΔSAT, mm	4.1 (2.2– 8.3)	64	6.8 (4.4–11.9)	89	153
VAT, mm	45.3 (40.5– 54.8)	77	40.8 (36.8–47.0)	103	180
ΔVAT, mm	11.4 (10.3)	55	8.2 (10.8)	69	124
cIMT, µm	430 (396– 491)	109	435 (376–495)	123	232
Distensibility coefficient, 10^{-3}/ kPa	40 (30–50)	101	40 (40–50)	117	218

© 2024 van der Linden IA et al. *JAMA Network Open.*

eFigure 2. Early-life risk factors for carotid intima-media thickness (cIMT) and carotid distensibility in adolescence

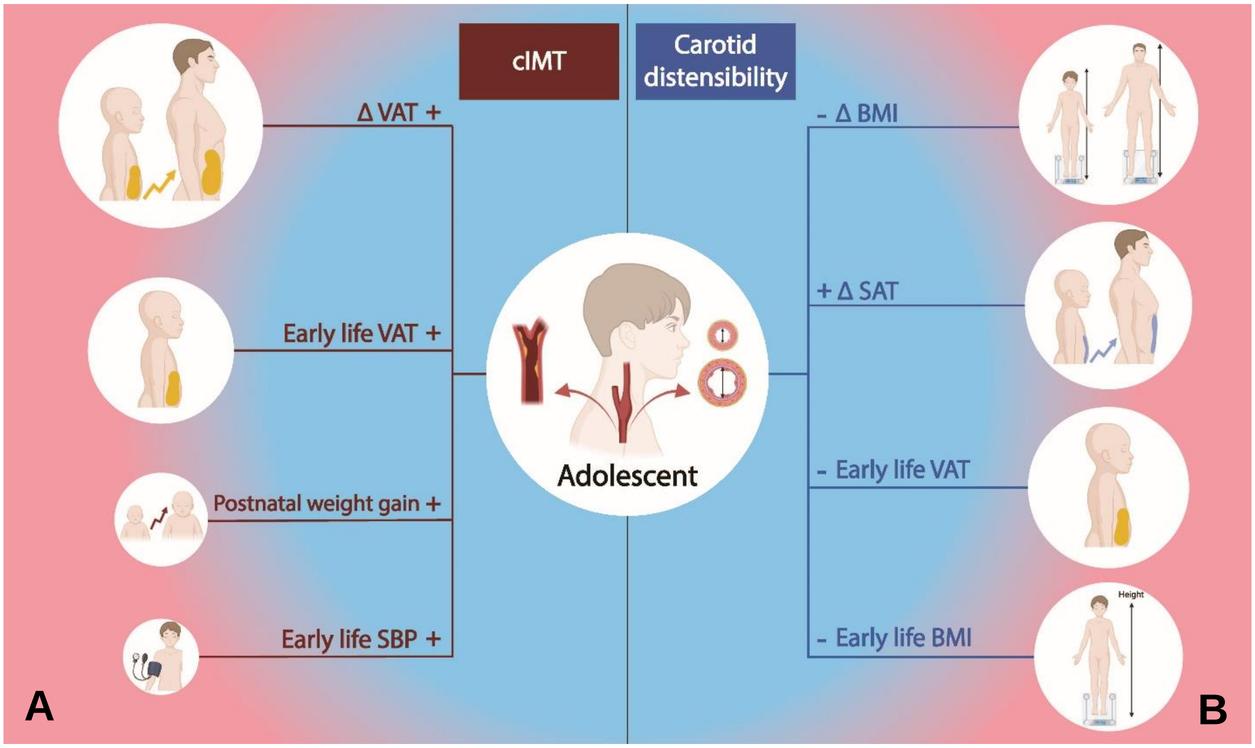

Summary of the study findings following assessment of cIMT (A) and carotid distensibility, assessed by the carotid distensibility coefficient (B). Risk factors with significant associations with the outcome measures are displayed in the white circles, scaled according to their effect size determined by the standardized beta coefficients. Plus signs (+) indicate a positive association and minus signs (-) indicate a negative association. VAT: abdominal visceral adipose tissue depth, SBP: systolic blood pressure, BMI: Body Mass Index, SAT: abdominal subcutaneous adipose tissue depth, "Δ" is the difference between the early life (5 years) and adolescent (12-16 years) value. Created with BioRender.com

© 2024 van der Linden IA et al. *JAMA Network Open.*
