# Supplementary material for: Early-Life Risk Factors for Carotid Intima-Media Thickness and Carotid Stiffness in Adolescence
Source: JAMA Netw Open. 2024 Sep 20;7(9):e2434699. doi: 10.1001/jamanetworkopen.2024.34699 (PMC11415786; doi:10.1001/jamanetworkopen.2024.34699)
Supplement: Supplement 2. — Data Sharing Statement [file jamanetwopen-e2434699-s002.pdf]

Data Sharing Statement

van der Linden. Early-Life Risk Factors for Carotid Intima-Media Thickness and Carotid Stiffness in Adolescence. *JAMA Netw Open*. Published September 20, 2024. doi:10.1001/jamanetworkopen.2024.34699

Data
Data available: No

Additional Information
Explanation for why data not available: The datasets for this article are available on reasonable requests, which should be directed to the corresponding author (Dr. Schipper). Any data sharing will be subject to meeting the Privacy Regulations of UMC Utrecht, the General Data Protection Regulation (GDPR) and the General Data Protection Regulation Implementation Act.
